# AACVD Synthesis and Characterization of Iron and Copper Oxides Modified ZnO Structured Films

**DOI:** 10.3390/nano10030471

**Published:** 2020-03-05

**Authors:** Martha Claros, Milena Setka, Yecid P. Jimenez, Stella Vallejos

**Affiliations:** 1 CEITEC—Central European Institute of Technology, Brno University of Technology, 61200 Brno, Czech Republic; Milena.Setka@ceitec.vutbr.cz (M.S.); stella.vallejos@imb-cnm.csic.es (S.V.); 2 Departamento de Ingeniería Química y Procesos de Minerales, Facultad de Ingeniería, Universidad de Antofagasta, 1270300 Antofagasta, Chile; yecid.jimenez@uantof.cl; 3 Instituto de Microelectrónica de Barcelona (IMB-CNM, CSIC), Campus UAB, 08193 Cerdanyola del Vallès, Barcelona, Spain

**Keywords:** AACVD, zinc oxide, iron oxide, copper oxide, structured films, water contact angle

## Abstract

Non-modified (ZnO) and modified (Fe_2_O_3_@ZnO and CuO@ZnO) structured films are deposited via aerosol assisted chemical vapor deposition. The surface modification of ZnO with iron or copper oxides is achieved in a second aerosol assisted chemical vapor deposition step and the characterization of morphology, structure, and surface of these new structured films is discussed. X-ray photoelectron spectrometry and X-ray diffraction corroborate the formation of ZnO, Fe_2_O_3_, and CuO and the electron microscopy images show the morphological and crystalline characteristics of these structured films. Static water contact angle measurements for these structured films indicate hydrophobic behavior with the modified structures showing higher contact angles compared to the non-modified films. Overall, results show that the modification of ZnO with iron or copper oxides enhances the hydrophobic behavior of the surface, increasing the contact angle of the water drops at the non-modified ZnO structures from 122° to 135° and 145° for Fe_2_O_3_@ZnO and CuO@ZnO, respectively. This is attributed to the different surface properties of the films including the morphology and chemical composition.

## 1. Introduction

Zinc oxide (ZnO) is a well-known n-type semiconducting material that possesses a wide band gap (3.37 eV) and large exciton binding energy (60 meV), hence its relevance in optoelectronics, photonics, and semiconducting devices [1]. Additionally, the tunable wetting properties of ZnO [2] has proved also attractive in the field of smart surfaces (e.g., self-cleaning surfaces for windows, solar cells, automobile windshields), micro/nano-fluidic systems, and sensors among others [3,4,5].

Overall, the aforementioned usages are strongly dependent on the chemical and physical properties of ZnO. Thus, according to the targeted application, transition metals (e.g., Mn, Co, Fe, Cu) and transition metal oxides (e.g., Cu_x_O_x_, Co_x_O_x_, Fe_x_O_x_) have been introduced as second-phase modifier materials in ZnO films to tune its electrical, optical and magnetic properties [4,6,7,8,9]. Similarly, the modification of ZnO with organic agents such as octadecylphosphonic acid (ODP) or alkanoic acids, as well as inorganic metal oxides such as cuprous oxide (Cu_2_O), has also been explored to improve its wettability [10,11,12,13] and sensing properties [14,15]. Hence, there is continuous interest in establishing new synthetic routes that allow tuning the properties of ZnO by the incorporation of second-phase materials.

Previously, several synthesis techniques have been employed to prepare non-modified and modified ZnO films with second-phase materials. Solution-based routes such as hydrothermal synthesis, sol-gel method, and chemical coprecipitation are among the most common procedures [16,17]. However, the integration of the materials synthetized by these techniques with silicon-based microfabrication processes (e.g., for electronics, sensors or microfluidics systems), is challenging and generally presents scalability issues. In contrast, chemical vapor deposition routes, including aerosol assisted chemical vapor deposition (AACVD), are good candidates to overcome this drawback, as these techniques are scalable and industrially attractive in silicon-based microfabrication processes. In particular, AACVD works at atmospheric pressure; therefore, the rates of deposition are typically magnitudes of orders higher than low-pressure processes, which impacts directly on the energy input required per gram of product. Moreover, AACVD is versatile and low-cost process, that allows the deposition of both thin films and micro/nanostructures via vapor-solid mechanism at moderated temperatures on different substrates [18,19]. A great variety of solvent precursor mixtures can be employed to change the morphology and size of the structures, since there is no need for the precursor to be volatile as in a regular CVD process [20]. At the same time, the process enables the modification of structures or thin films with second-phase materials in one or more subsequent steps [19,21,22].

AACVD has enabled previously the formation of ZnO structures via vapor-solid mechanism. Furthermore, it has been demonstrated that different ZnO morphologies (e.g., rods, needles, pyramides) may be deposited by simple changes in the AACVD operational parameters [5,18]. These morphological and therefore surface chemistry changes have shown to enhance properties such as wettability and gas sensing [5], hence the interest in tuning further the properties of AACVD ZnO structures and investigating its properties when modified with other metal oxides.

Therefore, here, it is presented the synthesis of ZnO structured films and their subsequent surface modification with Fe_2_O_3_ or CuO by AACVD. This work also evaluates and discusses the possible routes for AACVD deposition of Fe_2_O_3_ or CuO, as well as the morphological, chemical, and wetting properties of the synthetized structured films before and after surface modification.

## 2. Materials and Methods

### 2.1. Chemicals

Zinc (II) chloride of analytical grade (purity ≥ 0.98 in mass fraction) was purchased from Merck (KGaA, Darmstadt, Germany). Copper (II) nitrate hexahydrate and iron (III) chloride hexahydrate with a purity of >0.985 and >0.999 (mass fraction), respectively, were purchased from Sigma-Aldrich (GmbH, Albuch, Germany). Ethanol and acetone with purity > 0.999 (mass fraction) were purchased from Penta Chemicals (Prague, Czech Republic). All the reagents were used without further purification.

### 2.2. AACVD Synthesis

The AACVD synthesis was carried out in a horizontal hot-walled reactor, using nitrogen flow (Linde, oxygen free) and piezoelectric ultrasonic atomizer (Liquifog, Johnson Matthey, Redwitz, Germany).

Columnar zinc oxide (ZnO) structures were obtained via AACVD following the procedure presented before by Vallejos et al. [23]. Briefly, zinc chloride (50 mg) was dissolved in 5 mL of ethanol. The solution was converted into fine aerosol with the aid of the ultrasonic atomizer operating at 16 MHz. The aerosol formed was transferred by a nitrogen flow of 200 cm^3^·min^−1^ into the hot-wall AACVD reactor, which remained at a constant temperature of 723.15 K. The time to transport completely the solution into the reactor was typically 45 min. Silicon wafers were cut in pieces of 1 × 1 cm and used as substrates. For all the experiments, the substrates were cleaned thoroughly with plenty-deionized water and sonicated for 5 min in isopropanol. Then, the substrates were dried under nitrogen flow before placing them into the reactor.

The AACVD ZnO structures were modified with iron and copper oxides in a second step deposition. The conditions for the AACVD of iron and copper oxide were chosen by performing a systematic study of several parameters, including different solvents (acetone, methanol, ethanol) and temperatures (from 673.15 to 773.15 K). Hereafter, the optimized AACVD conditions used in this work are described. For the deposition of iron oxide, a solution of 3 mg of FeCl_3_·6H_2_O dissolved in 3 mL of acetone was prepared. This solution was placed in the aerosol generator and the mist was conducted by the nitrogen flow previously stablished to the reactor heated at a constant temperature of 723.15 K. The deposition lasted for 10 min until all the solution was completely consumed. Similar procedure was followed for the deposition of copper oxide. Typically, 3 mg of Cu(NO_3_)_2_·6H_2_O was dissolved in 3 mL of ethanol and placed in the aerosol generator. The temperature of the reactor was set at 723.15 K and the deposition lasted 25 min until all the solution was completely consumed. All the solutions were prepared only a few minutes prior to the synthesis.

### 2.3. Characterization

The morphology of the obtained nanostructures was observed by scanning electron microscopy (SEM, Tescan FE Mira II LMU, Brno, Czech Republic). A solid-state EDX detector (Bruker AXS, Inc., AZtec software package released by Oxford Instruments, Abingdon, UK) installed on the SEM performed the elemental line scans and mapping. TEM images were obtained with a scanning transmission electron microscopy (FEI Tecnai F20, 200 kV, Hillsboro, OR, USA) after removing the structures from the substrate and redeposit them on Cu grids. X-ray diffraction measurements were performed with a Bruker-AXS (LinxEye XE-T detector, KFL Cu 2K, λ (CuKα) = 1.541840 Å, Karlsruhe, Germany) operated at 40 KV and 40 mA. X-ray photoelectron spectroscopy (Kratos Axis Supra with monochromatic Al Kα X-ray radiation, emission current of 15 mA and hybrid lens mode, Manchester, UK) was used for the analysis of the surface. Wide and narrow spectra were measured with pass energy of 80 eV and 20 eV, respectively. XPS spectra were analyzed using CasaXPS software version 2.3.22. All spectra were calibrated using C 1s peaks with a fixed value of 284.7 eV. The Shirley algorithm was used to stablish the background of the spectra and the Gaussian–Lorentzian (GL) line shape was used to fit the XPS peaks. The Gibbs free energy for the proposed reactions were calculated by HSC chemistry 6 software (Outokumpu Research Oy, Pori, Finland).

Static water contact angle measurements were performed in a contact-angle measurement station (SEO Phoenix 300, Suwon City, Korea), with an automatic software controlled demi-water drop volume, set at 5 μL for each measure. The sample surfaces were gently cleaned with nitrogen flow before each measurement. Samples were tested in three different points, and the results presented here are an average of the three measures, each with 20 high-speed photographs. The static contact angle of the bare silicon substrates was examined before the AACVD of films. These tests registered a low water contact angle (52°) for the silicon surface. The drop photographs were acquired by a high-speed camera (Firewire digital camera) and the contact angles were analyzed by Surface-ware 8 contact angle analyzer software (Suwon City, Korea).

## 3. Results and Discussion

### 3.1. Zinc Oxide Films

**Figure 1:**
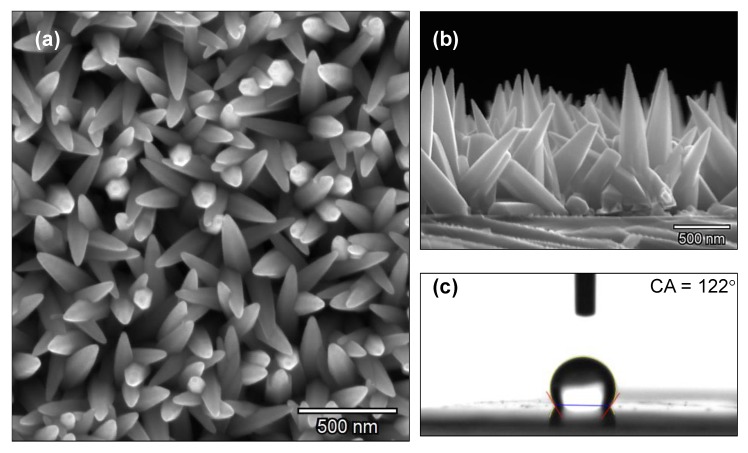
SEM images of the zinc oxide structures synthesized by AACVD on silicon substrate (**a**) top view; (**b**) cross section and (**c**) static water contact angle measurement.

The columnar ZnO structures deposited by AACVD are depicted in Figure 1a,b. SEM images indicate that the obtained structures have a well-defined rod-like morphology with spear-shape ending. The average length of the roads was of ~1.2 µm and a mid-height diameter of ~80 nm, the base of the structures is ~300 nm, whereas the smallest diameter at the top is ~22 nm. After the ZnO deposition, the silicon substrates presented a homogeneous grey to white opaque color, in contrast to the silver shining bare silicon substrate. The measured static water contact angle for the pure ZnO film was registered as 122° (see Figure 1c).

The chemical composition of the deposited products was analyzed by EDX and XPS. The EDX analysis indicated that the resulted nanostructures were composed by zinc and oxygen as major components; additionally, the presence of chlorine was detected as a product of unreacted precursor (0.9 wt.%).

**Figure 2:**
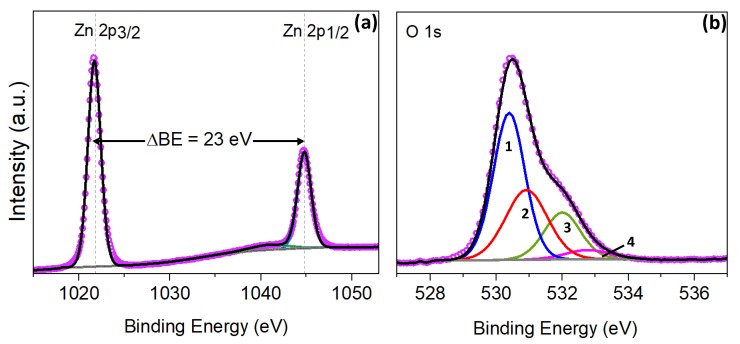
(**a**) Zn 2p and (**b**) O 1s core level XPS spectra of the non-modified ZnO films. Pink hollow dots show the XPS raw data, the black solid line corresponds to the envelope-fitting curve, and the colored solid lines to the components.

Further studies of the ZnO deposited structures by XPS analysis in Figure 2a shows the characteristic Zn 2p doublet peaks corresponding to Zn^2+^ oxidation state [17,23,24]. The components centered at binding energies (BE) of 1021.8 eV and 1044.9 eV correspond to Zn 2p_3/2_ and 2p_1/2_, respectively, while a shake-up is located at 1040.2 eV. These Zn 2p_3/2_ and 2p_1/2_ core level binding energies are slightly shifted compared to other reports [17,23,24,25,26,27], however, the separation between them is constant and equals to 23 eV, in agreement with the literature as shown in Table 1.

The XPS spectrum of the O 1s region present an asymmetric peak, indicating the presence of different oxygen species. The curve was fitted to four distinctively GL components (1, 2, 3 and 4, as shown in Figure 2b). The peak 1 centered at 530.4 eV is attributed to O^2−^ ions in the Zn-O bonding of the wurtzite structure of Zn^2+^ [17,28,29], whereas the peak 2 situated at 530.9 eV is assigned to O^2−^ state of oxygen defects or vacancies, supporting the formation of the non-stoichiometric ZnO. Finally, the peaks 3 and 4 at 532.0 eV and 532.8 eV, respectively, are typically related to weak bonds of oxygen on the surface, such as OH groups [29,30,31].

### 3.2. Iron Modified Zinc Oxide Films

**Figure 3:**
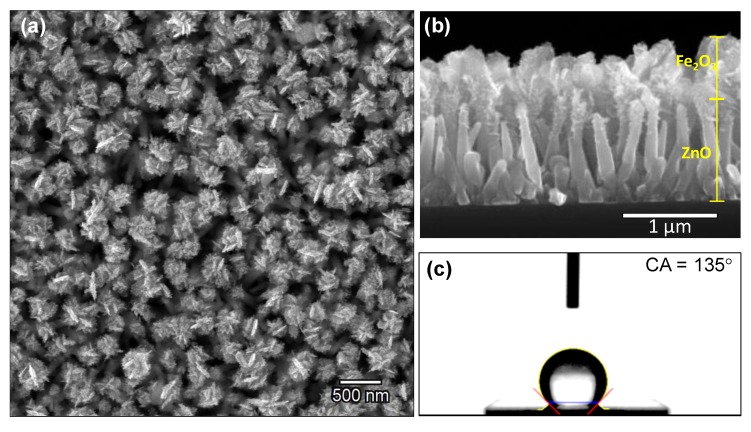
SEM images of Fe_2_O_3_@ZnO structures (**a**) top view; (**b**) cross section and (**c**) static water angle measurement.

The ZnO structures modified with iron oxide are depicted in Figure 3a,b. These SEM images show the morphology of the ZnO structured film and the iron oxide deposited in the second deposition step. The cross-section SEM image in Figure 3b displays the irregular iron oxide layer of aggregated particles with sharp edges, resembling a nanoflake-like morphology. The total thickness of these structures formed over the ZnO rods is about 200 nm. This corresponds with the thickness measured for an AACVD deposited iron oxide on bare silicon substrate. The static water contact angle of these samples is 135° as displayed in Figure 3c.

**Figure 4:**
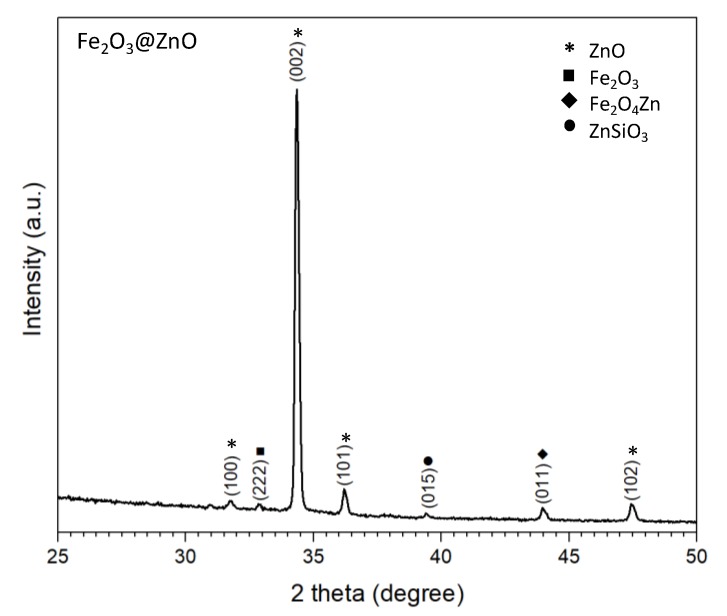
XRD diffraction pattern of the Fe_2_O_3_@ZnO films.

The XRD diffraction pattern of the Fe_2_O_3_@ZnO nanostructured surfaces is displayed in Figure 4. The pattern shows the presence of a hexagonal ZnO phase, with a high intensity peak at 34.4° 2θ, that indicate a preferred orientation in the (002) direction (P63mc space group, ICCD card No. 50664); this is in good agreement with our previous results for bare ZnO deposited via AACVD [23]. In addition, the pattern indicates the presence of three other phases, represented by diffractions with less intensity as they may be related to smaller amounts of particles compared to the ZnO. Thus, the (222) plane of the cubic Fe_2_O_3_ (Ia-3 space group, ICSD Card No. 108905) is visible at 33.0° 2θ, in agreement with previous Fe_2_O_3_ structures deposited by AACVD [32]. The patterns also suggest the presence of other planes related to cubic Fe_2_O_4_Zn (Fd-3m space group, ICSD Card No. 91940) and rhombohedral ZnSiO_3_ (R-3 space group, ICSD Card No. 340575) most likely present at the interfaces of Fe_2_O_3_/ZnO and ZnO/Si (from substrate), respectively.

**Figure 5:**
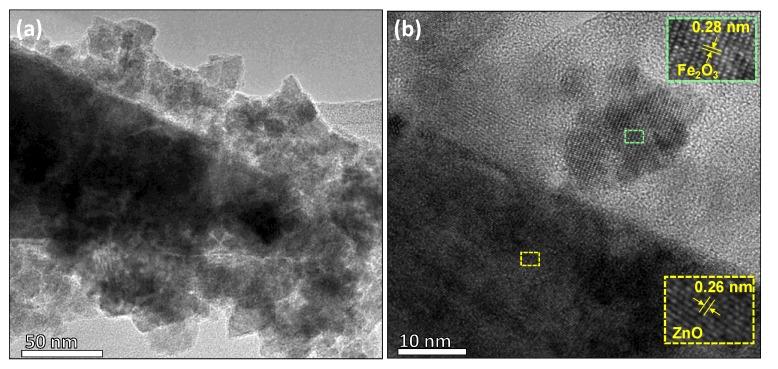
(**a**) Low and (**b**) high resolution TEM image for the Fe_2_O_3_@ZnO structures. The insets display the plane spacing of Fe_2_O_3_ and ZnO. The colour code corresponds to the areas indicated in the TEM image from which the insets were taken.

The structured films were also analysed by TEM after the deposition of iron oxide over the zinc oxide structures (Figure 5a). The TEM images reveal the zinc oxide rod surface, covered by agglomerated iron oxide particles. The HRTEM image of this particle displays well-ordered atomic array (Figure 5b) with the planar spacing of 0.26 nm approximately; this is consistent with the (002) internal lattice spacing of the hexagonal ZnO, also found in the XRD diffraction pattern (d = 0.2602 nm, ICCD card No. 50664). The HRTEM image also shows the presence of a second phase with a planar spacing of approximately 0.28 nm, that is in agreement with the internal lattice spacing of the (222) plane of the cubic phase of Fe_2_O_3_ (d = 0.271 nm, ICSD card No. 108905) identified in the XRD pattern shown above.

To determine the surface chemical composition, EDX analysis was performed. This analysis showed as a result zinc and iron as major components, as well as the presence of chlorine traces (0.8 wt.%), which might be related to unreacted precursor from zinc and/or iron during deposition.

**Figure 6:**
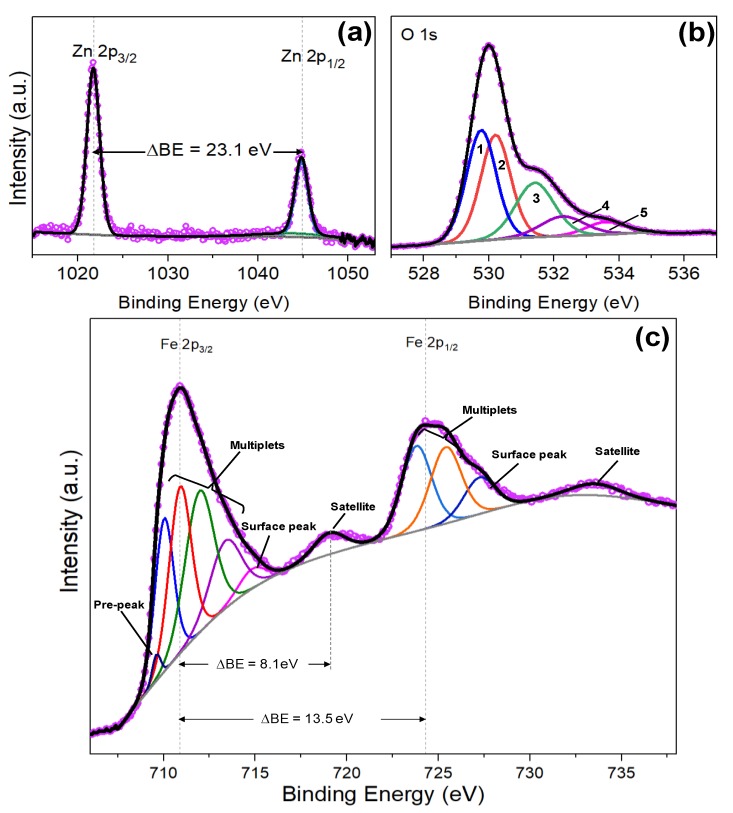
(**a**) Zn 2p, (**b**) O1s and (**c**) Fe 2p core levels XPS spectra of the Fe_2_O_3_@ZnO films. Pink hollow dots represent the raw data, the black solid line corresponds to the envelope/fitting curve, and the colored curves to the deconvoluted components.

Detailed chemical composition was studied by XPS analysis. The Zn 2p core level spectrum is depicted in Figure 6a. As seen above for the non-modified ZnO structures (Figure 2a), the characteristic Zn 2p doublet is also present, although it shows a slight shift to higher binding energies. Thus, the Zn 2p core level peaks appear at 1021.9 eV for Zn 2p_2/3_ and 1045.1 eV for Zn 2p_1/2_, the separation between both peaks is equals to 23.1 eV, in agreement with the literature [26] and the results shown for the bare ZnO.

The O 1s and Fe 2p core level spectra are depicted in Figure 6b,c, respectively. An asymmetric peak is observed for O 1s spectrum, and after deconvolution, five GL components are determined. Peaks 1 at 529.7 eV and peak 2 at 530.1 eV are usually related to the O^2−^ bonding with metals [17], i.e., Fe-O and Zn-O for the present study, whereas peak 3 at 531.4 eV is associated with the oxygen vacancies as noticed above for the non-modified ZnO structures. Peak 4 situated at 532.5 eV is connected with the contribution of surface OH groups and possible Zn-Fe-O bond [33], and peak 5 at 533.6 eV is associated with the presence of iron oxide-hydroxide (FeO(OH)) and H_2_O adsorbed in the surface of the nanostructures, as reported in literature [34,35,36].

The Fe 2p core level spectrum shown in Figure 6c, contains two main peaks at 710.8 and 724.4 eV corresponding to Fe 2p_3/2_ and Fe 2p_1/2_, respectively and two satellite peaks at 718.9 eV and 733.4 eV. The difference binding energy between Fe 2p_3/2_ and Fe 2p_1/2_ peaks is 13.5 eV, which results from spin-orbit (j-j) coupling and is in accordance with literature values [31,37]. The Fe 2p_3/2_ peak was deconvoluted and fitted to six GL peaks. The distribution of these peaks show a characteristic pre-peak at 709.6 eV, followed by multiple splitting of the four peaks and finally a surface peak at 714.8 eV, which is consistent with the data found in literature for the Fe^3+^ ion oxidation state [35,38], and rules out the presence of Fe^2+^ species. This is also corroborated by the satellite peak typically associated with Fe_2_O_3_, which is located at a higher binding energy (8.1 eV) than the main Fe 2p_3/2_ peak, as reported previously in literature (8 eV) [35,36,37,38,39,40].

### 3.3. Copper Modified Zinc Oxide Films

**Figure 7:**
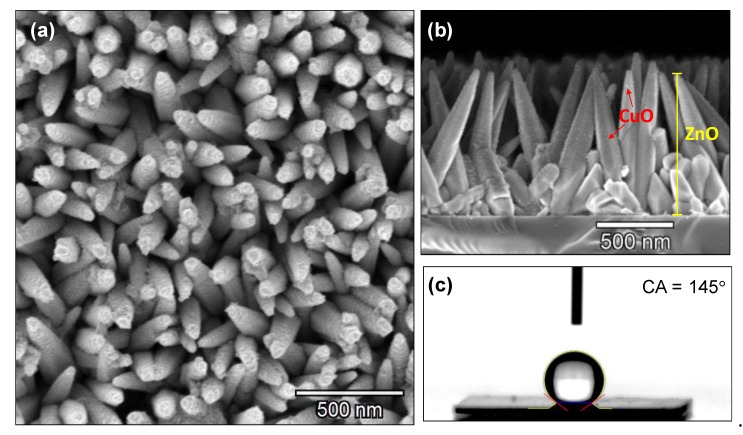
SEM images of CuO@ZnO structures (**a**) top view; (**b**) cross section and (**c**) static water contact angle.

Figure 7a,b, show the SEM images of the Cu-modified ZnO structures. These images show the copper oxide deposited as spherical-like particles (as shown in Figure 7b) that tend to aggregate on the top of the columnar ZnO structures and disperse evenly along their side walls. The contact angle determined for these films is 145° as displayed in Figure 7c.

**Figure 8:**
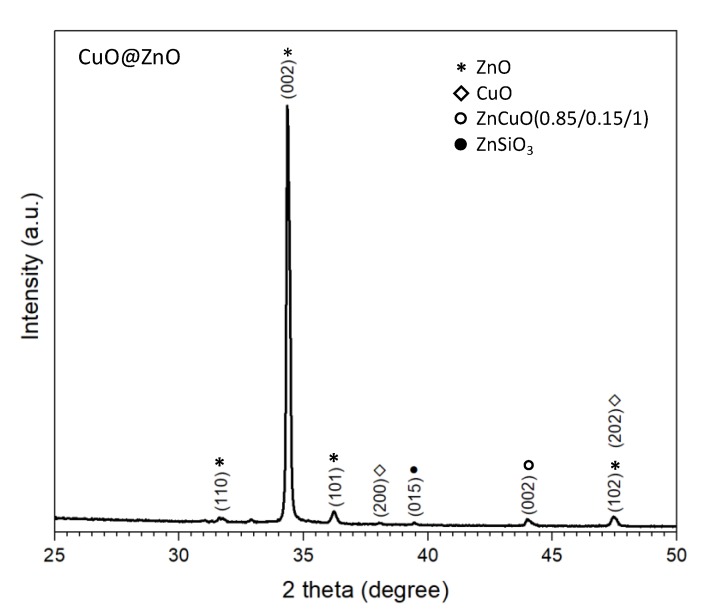
XRD diffraction pattern of the CuO@ZnO films.

The XRD diffraction pattern of the CuO@ZnO films is presented in Figure 8. Similar to previous case (i.e., Fe_2_O_3_@ZnO), the pattern shows intense diffraction peaks that correspond to the hexagonal zinc oxide phase and indicate a preferential orientation in the (002) plane. In addition, the pattern shows three other phases. The first connected with the monoclinic copper (II) oxide phase (C12/c1 space group, ICSD Card No. 160630) in which the (200) plane and (202) plane are observed, the later overlapped with the (102) plane of hexagonal ZnO. The second and third attributed to the cubic ZnCuO (0.85/0.15/1) compound (Fm-3m space group, ICSD Card No. 181023) and the ZnSiO_3_ (R-3 space group, ICSD Card No. 340575), as in previous case, most likely present at the interfaces of CuO/ZnO and ZnO/Si (from substrate), respectively.

**Figure 9:**
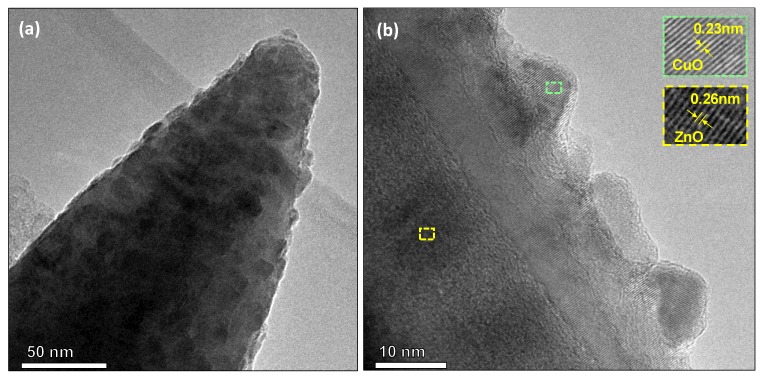
(**a**) Low and (**b**) high resolution TEM images for the copper oxide modified zinc oxide rods. The insets display the plane spacing of CuO and ZnO. The colour code corresponds to the areas indicated in the TEM image from which the insets were taken.

To get better insight into the structures formed by AACVD, TEM images were carried out. Figure 9, shows the columnar zinc oxide structure covered by the copper oxide particles. As can be seen, the particles with spherical morphologies are well-distributed over the rod surface. The size of the particles is between 8 and 20 nm (recorded for a total population 25 particles). The HRTEM images show a well-ordered planar atomic array with planar spacing of approximately 0.26 nm and 0.23 nm. The first corresponds to the internal lattice spacing of the (0020) plane of the hexagonal ZnO phase (d = 0.2602 nm, ICCD card No. 50664) identified by XRD, whereas the second is associated to the (200) plane of the monoclinic CuO phase (d = 0.2362 nm, ICSD Card No. 160630), also determined in the XRD analysis.

The chemical composition of the film was first evaluated by EDX analysis. This analysis revealed the presence of copper along with zinc as the major component. Similar to the previous cases, for ZnO and Fe_2_O_3_@ZnO, chlorine traces (0.7 wt.%) were also detected. 

**Figure 10:**
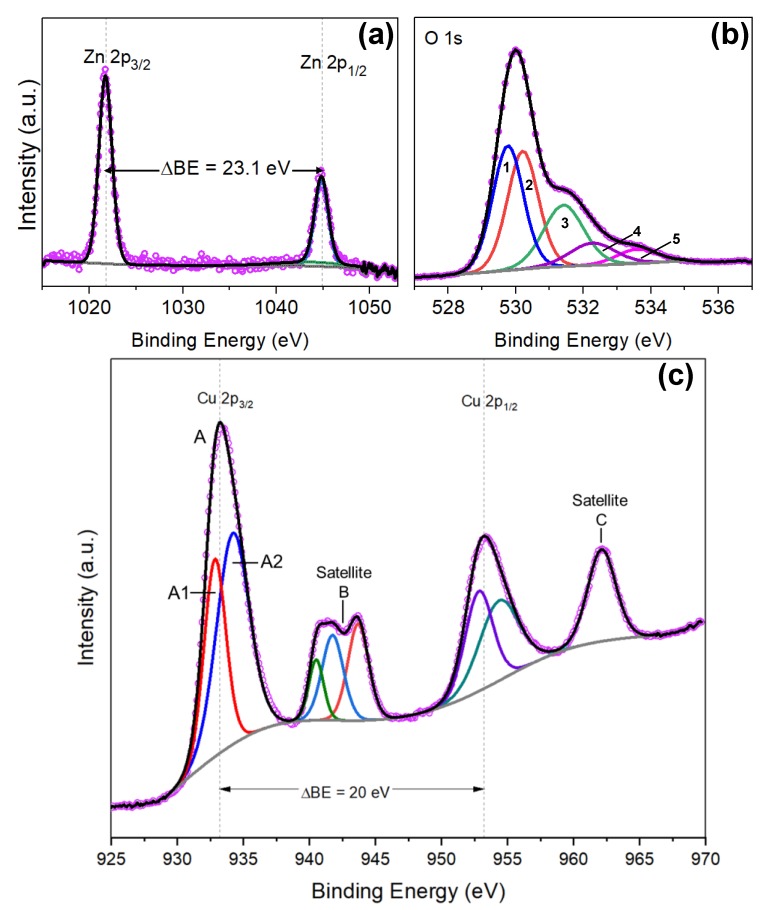
(**a**) Zn 2p, (**b**) O 1s and (**c**) Cu 2p core levels XPS spectra for CuO@ZnO films. Pink hollow dots represent the raw data, the black solid line corresponds to the envelope/fitting curve, and the colored curves to the deconvoluted components.

A more detailed chemical composition was performed by XPS analysis. The XPS spectrum for the Zn 2p is shown in Figure 10a. The Zn 2p characteristic doublet peaks are slightly shifted to higher binding energies (1021.9 eV for Zn 2p_3/2_ and 1045.0 eV for Zn 2p_1/2_) as compared to the Zn 2p peaks in the bare ZnO (Figure 2a). As seen before, the separation between these two main peaks remains at 23.1 eV, consistent with the literature [26].

The O 1s XPS spectrum presents an asymmetric curve as in the afore cases. Five distinguishable curves after deconvolution were determined, as depicted in Figure 10b. In agreement with the literature [41], peak 1 at 529.5 eV is associated with the bonding between copper and O^2−^ (Cu-O), whereas peak 2 located at 530.7 eV is related to the Zn-O wurtzite lattice bond, as established above. Peak 3 at a binding energy of 531.7 eV is assigned to the oxygen deficiency regions, i.e., oxygen vacancies. Finally, peak 4 at 532.7 eV, and peak 5 at 533.9 eV may be associated with chemisorbed oxygen, hydroxides, and H_2_O on the surface of the Cu-modified ZnO structures [33,42].

The Cu 2p core level spectrum show the characteristic peaks at binding energies of 933.2 eV for Cu 2p_3/2_ and 953.2 eV for Cu 2p_1/2_, with a difference between both peaks of 20 eV (Figure 10c). Along the main peaks, two satellite peaks, B and C, are found. The peak B consists of three components at 940.5 eV, 941.1 eV and 943.5 eV (B peaks), whereas the peak C is described by one component at 962.1 eV.

### 3.4. Discussion

Results show the formation of ZnO structures via aerosol assisted chemical vapor deposition, and the surface modification of these structures by Fe_2_O_3_ and CuO in a second step using the same synthetic method. These structured films demonstrated visible morphological and chemical composition changes respect to the bare ZnO structures as well as changes in the surface properties including the wettability properties.

The surface chemical composition of the synthesized films was studied using EDX and XPS analysis. EDX analysis showed a similar content of chlorine in the films (0.9 wt.%, 0.8 wt.%, and 0.7 wt.%, for bare the ZnO, Fe_2_O_3_@ZnO, and CuO@ZnO, respectively). Although this low chlorine contents can influence the functional properties of the surface in certain applications (e.g., chemical sensing), to the best of our knowledge, there is no evidence in which these traces have shown significant effects on the contact angle of demi-water drops. In this context and due to the similar chlorine contents in the tested samples, we rule out the influence of this ion on the wetting properties of the structured films. For the bare ZnO rods, the XPS analysis showed the characteristic peaks for the Zn^2+^ state, i.e., Zn 2p_3/2_ and 2p_1/2_ core level binding energies. Previous reports have found that for a stoichiometric ZnO compound, the 2p_3/2_ and 2p_1/2_ core level XPS peaks are located at 1022.0 eV and 1045 eV, respectively [23,25,27]. However, the XPS records of the AACVD structures presented an overall shift to lower binding energies, which is usually related to the formation of nonstoichiometric ZnO due to oxygen vacancies induced by the structural size reduction [43]. This oxygen deficit is supported by the O 1s spectrum, which after the deconvolution presents a component linked to the oxygen vacancies (component 2 in Figure 2b).

After the second deposition step by AACVD, the spear pointing ending structures of the bare ZnO are covered by the Fe_2_O flake-like structure, as evidenced in the SEM and TEM images (Figure 3 and Figure 5). The incorporation of these iron compound also altered the chemical surface composition. XPS spectra analysis indicate that Zn 2p doublet are shifted to higher binding energy compared to bare ZnO; this may be related to the incorporation of Fe^3+^ ion into the ZnO lattice, as noticed previously in the literature [31]. In addition, the deconvolution of O 1s spectra shows a component situated at 532.5 eV that is not only related to surface OH groups, but also to a possible Zn-Fe-O bond [33]. This is consistent with the XRD diffractions, in which it was registered diffractions liked to the Franklinite (Fe_2_O_4_Zn) compound (Figure 4).

The results obtained from the XPS analysis through deconvolution and fitting, suggest the complete oxidation of the precursor into Fe_2_O_3_ during the AACVD synthesis. Usually the chemical reactions involved in the synthesis process are not well defined, and in an attempt to understand this process, two consecutive chemical reactions are proposed as possible routes for the AACVD formation of Fe_2_O_3_ from the iron precursor dissolved in acetone at a temperature of 723.15 K. The estimation of the Gibbs free energy (ΔG) for the following reactions were calculated by HSC chemistry software (see materials and methods section). The first reaction (1) may involve the formation of FeO(OH) and the release of gaseous species such as chloromethane, hydrogen, and carbon dioxide, whereas the subsequent second reaction (2) may involve the formation of Fe_2_O_3_ and release of water and oxygen.

FeCl_3_·6H_2_O + 2C_3_H_6_O(a) → FeO(OH) + 3CH_3_Cl(g) + 7H_2_(g) + 3CO_2_(g) (1)

ΔG = −97.18 kcal at 723.15 K

2FeO(OH) + 2O_2_(g) → Fe_2_O_3_ + H_2_O(g) + 2O_2_(g) (2)

ΔG = −13.05 kcal at 723.15 K

Notice that for both reactions the calculated ΔG is negative (−97.18 kcal and −13.05 kcal for the first and second reaction, respectively), indicating that the reaction enthalpy (ΔH) is lower than the entropy (ΔS) (i.e., the ΔS of the products is greater than the reactants ΔS) therefore the proposed reactions are thermodynamically feasible.

Similar to this analysis, the incorporation of copper oxide in the ZnO structures was studied. As seen in the SEM and TEM images, the deposition of the copper oxide in a AACVD second step, modifies the morphology of the bare ZnO, as well as the surface chemical composition. XPS analysis showed the Zn 2p characteristic doublet peaks are slightly shifted to higher binding energies (1021.9 eV and 1045.0 eV for Zn 2p_3/2_ and Zn 2p_1/2_, respectively) as compared to the Zn 2p peaks in the bare ZnO (Figure 2a). This shifting of the binding energy could be related to the Zn-O bond length change, which is likely connected with the incorporation and/or substitution of Cu^2+^ ion into the ZnO lattice [14,33,44]. The diffraction connected with the ZnCuO compound (Figure 8) also corroborate the presence of Zn-Cu bonding. The component at 529.5 eV (peak 1, Figure 10b) in the O 1s spectra of the CuO@ZnO films also suggest the presence of the Cu-O bond in the film [45].

Due to typical formation of different copper oxidation states, i.e. Cu^0^, Cu^+^ and Cu^2+^, during oxidation, the Cu 2p XPS spectrum was analyzed to determine the main specie in the structured film. To this end, it was employed the method described by Jasieniak and Gerson [26], which relates the deconvoluted fitted areas of the Cu 2p_3/2_ peak and its closest satellite peak. To calculate the percentage of the components, the fitted areas of Cu 2p_3/2_ peak (components A1 and A2) and the nearest shake-up peak (satellite B) were considered, as described elsewhere [45]. Shake-up peaks are typically generated when the outgoing photoelectron interacts simultaneously with a valence electron and excites it to a higher-energy level. In this process, the kinetic energy of the photoelectrons is slightly reduced giving a satellite structure in a lower energy (higher in the binding energy scale) than the core level position [14,39,45]. For the present results, the deconvolution of the Cu 2p_3/2_ XPS peak (Figure 10c), indicate the co-existence of a mixed oxidation states, and thus the peak A1 at 932.8 eV might be related to Cu^2+^, and peak A2 at 934.3 eV might be related to Cu^0^ and Cu^+^ contribution. The presence of satellites shake-up peaks in B (components at 940.5 eV, 941.1 eV and 943.5 eV), are associated with Cu^2+^ oxidation state since these peak is characteristic of a partially filled d-orbital (d^9^) and is not present in the d^10^ Cu^+^ spectra. Therefore, the estimation of the content of (Cu^0^ + Cu^+^) and Cu^2+^ is approximately 17% and 83%, respectively. These suggest that the compound deposited on the ZnO structures corresponds mainly to CuO.

In a like manner to the former case, it is proposed that the AACVD formation of CuO occurs through two consecutive chemical reactions (Equations (3) and (4)), that are possible routes for copper nitrate oxidation in the presence of ethanol at high temperature (T = 723.15 K). Under these temperature conditions, it was found that the Gibbs free energy is negative for both possible reactions (−531.20 kcal and −8.31 kcal for reactions 3 and 4, respectively). This is related to enthalpy reactions (ΔH) that are lower than the entropy (ΔS), and thus the entropy of the products is greater than the reactants entropy making the reactions thermodynamically possible.

6Cu(NO_3_)_2_·6H_2_O + C_2_H_5_OH(a) → 6Cu(OH)_2_ + 12NO_2_(g) + 2CO_2_(g) + 15H_2_O(g) (3)

ΔG = −531.20 kcal

Cu(OH)_2_ + O_2_(g) → CuO + O_2_(g) + H_2_O(g) (4)

ΔG = −8.31 kcal

The oxidation of the Cu(OH)_2_ in air (proposed in Equation (4)) is experimentally observed when the reactor chamber is open (immediately after deposition without temperature alteration) as the color of the film change instantaneously from dark orange to dark brown-black.

The alterations in morphology and surface composition of the ZnO structures also showed an effect in the wetting properties of the structured films. We noticed that the static water CA of the films increased from 122° (bare ZnO) to 134° (Fe_2_O_3_@ZnO) and 145° (CuO@ZnO). These results are consistent with the literature, which showed previously the possibility of tuning the wetting capability of a surface towards more hydrophobic (CA > 90°) or hydrophilic (CA < 90°) characteristics [46] by modifying the surface morphology and chemical composition of a surface [47,48].

The different wetting properties of the modified films (Fe_2_O_3_@ZnO and CuO@ZnO) could be attributed in part to the surface morphology, which suggests a favorable Cassie-Baxter regime with a CA exceeding the 120° [49]. In addition, the wetting properties could also be attributed to the surface chemistry of the films, in particular to the oxygen vacancies as noticed previously in the literature [50]. The results obtained from the deconvolution of the O 1s XPS spectrum made through conventional fitting procedure indicate that all samples (i.e., ZnO, Fe_2_O_3_@ZnO and CuO@ZnO) present a component associated with O^−^ and O_2_^−^ ions in the oxygen deficient regions –oxygen vacancies– (O_V_). A comparison of the atomic percentage concentration (at.%) of these species (that generally is the representation in percentage of the intensity of a type of oxygen respect to the other oxygen species at the surface) indicates that the O_V_ at the non-modified ZnO is higher (30.2 at.%) compared to Fe_2_O_3_@ZnO (20.7 at.%) and CuO@ZnO (18.6 at.%). Also, it is noticed that the O_V_ concentration of Fe_2_O_3_@ZnO is higher compared to CuO@ZnO, this could be related to the ion size and charge of the iron ion, as Fe^3+^ (0.63 Å) ion is smaller than Cu^2+^ (0.73 Å) ion, and the extra positive charge of Fe^3+^ compared with Cu^2+^ attracts more oxygen to the lattice, and consequently the incorporation of Fe^3+^ in ZnO structures reduces the surface oxygen compared to Cu^2+^ [44,51].

Previously, it has been suggested that a preferred dissociation adsorption of water molecules takes place, with the formation of hydroxyl groups (OH^−^) in surfaces with oxygen vacancies. Therefore, when the oxygen vacancies concentration at the surface is low is expected that the film surface became more hydrophobic [50,52]. As a result of the analysis, the oxygen vacancies decrease in the following order ZnO > Fe_2_O_3_@ZnO > CuO@ZnO and water CA increases correspondingly 122° < 135° < 145°. Therefore, the decrease in oxygen vacancies concentration that is achieved with the deposition of Fe_2_O_3_ and CuO in the ZnO structured films increases the contact angle. Overall, these results demonstrate that the modification of ZnO with iron and copper oxides modifies the wetting properties, increasing the hydrophobicity of the films. This behavior is correlated in part with the surface morphology and the oxygen deficiency at the surface of each film.

## 4. Conclusions

The synthesis of ZnO structures in one step, and their surface modification with either Fe_2_O_3_ or CuO in a second step was achieved using the AACVD method. The AACVD conditions employed proved to be reliable and repeatable. SEM and TEM images of the non-modified ZnO films revealed an initial spear-ending like morphology, which after the deposition of Fe_2_O_3_ or CuO was covered by flake-like structures or spherical nanoparticles, respectively. The formation of crystalline ZnO, Fe_2_O_3_, and CuO structures was determined by X-ray diffraction and HRTEM analysis. Deconvolution and fitting of the XPS spectra recorded on the Fe_2_O_3_@ZnO films showed the presence of Fe^3+^ oxidation state, indicating the formation of Fe_2_O_3_. Similarly, the XPS of the CuO@ZnO films showed the presence of (Cu^0^ + Cu^+^) and Cu^2+^ co-existing oxidation states, with Cu^2+^ as the major component, indicating that the particles formed are composed mainly of CuO. Wettability studies by static water contact angle of these films show that the modification of the ZnO surface with iron and copper oxides increases the hydrophobicity of the ZnO surfaces, from 122° for bare ZnO to 135° and 145° for Fe_2_O_3_@ZnO and CuO@ZnO, respectively. Results lead to the conclusion that this behavior is related to both morphological changes and chemical surface alterations.

## Figures and Tables

**Table 1:** Comparative table showing the Zn 2p_3/2_ and Zn 2p_1/2_ binding energy values from the literature.

Structure Type	Size (nm)	Binding Energy Zn 2p_3/2_ (eV)	Binding Energy Zn 2p_1/2_ (eV)	Zn 2p_1/2_-Zn2p_3/2_ Splitting (eV)	Ref
Nanoparticle	~30	1020.7	1043.7	23.0	[17]
Nanoflowers	100–250 ^a^	1020.8	1043.8	23.0	[17]
Nanorods	45–96 ^b^	1021.2	1044.2	23.0	[17]
Nanosheets	10 ^c^	1022.0	1045.0	23.0	[25]
Nanoplates	10–15^c^	1021.1	NR	NR	[24]
Rods	~380 ^b^	1022.0	1045.0	23.0	[23]
Bulk film	NR	1021.0	1044.1	23.1	[26]
Bulk film	NR	1022.0	1045.0	23.0	[27]
Spear-like rods	~80 ^d^	1021.8	1044.8	23.0	This work

^a^ Tip-base relation; ^b^ Diameter; ^c^ Thickness; ^d^ Mid-height diameter, NR: not reported.
